# Management paradigm for ovarian neuroendocrine carcinoma: a systematic review

**DOI:** 10.1186/s13048-025-01701-7

**Published:** 2025-06-09

**Authors:** Kemala Isnainiasih Mantilidewi, Gatot Nyarumenteng Adhipurnawan Winarno, Ali Budi Harsono, Dodi Suardi, Yudi Mulyana Hidayat, Andi Kurniadi, Siti Salima, Febia Erfiandi, Aini Sofa Haniah, Nirmala Chandralega Kampan

**Affiliations:** 1 Department of Obstetrics and Gynecology, Faculty of Medicine, Universitas Padjadjaran, Dr. Hasan Sadikin General Hospital, Prof Eykman Street No 38, Bandung, 40161 Indonesia; 2 Gynae-Oncology Unit, Department of Obstetrics and Gynaecology, Hospital Canselor Tuanku Muhriz, Faculty of Medicine, Universiti Kebangsaan Malaysia, Kuala Lumpur, Malaysia

**Keywords:** Ovary, Neuroendocrine carcinoma, Treatment, Outcomes

## Abstract

**Introduction:**

Neuroendocrine neoplasms (NENs) of the female genital tract are rare, comprising only 1–2% of gynecologic tumors, with ovarian neuroendocrine carcinoma (O-NEC) accounting for less than 1% of all ovarian cancers. Despite its rarity, O-NEC is a highly aggressive tumor with poor prognosis and significant diagnostic complexity, warranting focused clinical attention and demand greater awareness to improve diagnostic and therapeutic strategies.

**Methods:**

This systematic review analyzed management paradigm for O-NEC through a comprehensive search on the databases PubMed, Science Direct, Wiley, Springer Link, Google Scholar and Cochrane Central Register of Controlled Trials that was performed on August 1st, 2024.

**Results:**

A comprehensive search on August 1st, 2024, identified 21 eligible studies (6 retrospective cohorts, 12 case reports, 3 case series), encompassing 923 cases of O-NEC. The most common subtypes were small-cell (40%) and large-cell (39.8%) NEC. Most patients presented with advanced-stage (Stage III–IV: 52%). Immunohistochemical markers included synaptophysin (84%), chromogranin A (64.2%), CD56 (69%), and NSE (77.7%). Treatment varied from surgery alone (35%) or surgery plus chemotherapy (32%) being most common. No standardized regimen of chemotherapy was identified with etoposide/cisplatin and paclitaxel/carboplatin were most frequently used. Survival outcomes were poor, with median overall survival ranging from 11 to 23.5 months. The stage at diagnosis was a crucial prognostic factor.

**Conclusions:**

The O-NEC is a rare, heterogeneous malignancy with diverse histopathology, variable immunohistochemical profiles, and generally poor prognosis. Early-stage disease may be managed with surgery alone, while advanced stages require multimodal treatment including surgery with adjuvant platinum-based chemotherapy. Due to limited cases and predominantly retrospective data, standardized diagnostic and treatment protocols are lacking. Prospective multicenter studies and centralized registries are needed to improve understanding and patient outcomes.

**Supplementary Information:**

The online version contains supplementary material available at 10.1186/s13048-025-01701-7.

## Introduction

Neuroendocrine neoplasms (NENs) represent a diverse group of epithelial tumors that arise from neuroendocrine cells distributed throughout the body. These tumor can develop in various organs, including gastrointestinal tract, lungs, and genital tract [1]. The NENs range from well-differentiated, slow-growing neuroendocrine tumors (NETs) to highly aggressive, poorly differentiated neuroendocrine carcinomas (NECs) [2]. Although NENs can develop in almost any organ and share similar morphological and immunophenotypic traits, they also display notable site-specific features [1]. A key distinction between NETs and NECs lies in their morphology, proliferation rates (such as Ki67 index), and underlying molecular pathways. Neuroendocrine tumors (NETs) typically harbor alterations in genes like MEN1 (Multiple Endocrine Neoplasia type 1), VHL (Von Hippel–Lindau gene), and TSC1/2 (genes involved in tuberous sclerosis complex), and show activation of the PI3K/mTOR signaling pathway, whereas NECs are commonly associated with inactivation of TP53 and RB1, mirroring the molecular profile of non-neuroendocrine carcinomas arising from the same primary sites. As a result, NETs and NECs are now regarded as separate clinicopathological entities, with significant differences in diagnosis, prognosis, and therapeutic approach [3]. 

Female genital tract NENs are uncommon, constituting just 1 to 2% of gynecological tumors, and are categorized by organ site, including the cervix, ovaries, uterus, vulva, and vagina [4]. Crane et al. reported the distribution of gynecologic neuroendocrine neoplasms as follows: 54% were located in the cervix, 24% in the uterine corpus, 16% in the ovary or fallopian tube, 5% in the vagina, and 1% in the vulva. Among gynecologic sites, NETs are most commonly found in the ovary, while NECs primarily occur in the cervix [5]. However, NECs can also occur in the ovaries, though much less frequently. Ovarian neuroendocrine carcinoma (O-NEC), accounting for less than 1% of all ovarian malignancies, is considered a highly aggressive subtype of ovarian cancer [6]. 

The classification of ovarian NENs undergo changes over time. According to the 2022 WHO classification, ovarian NENs are divided into two main groups by histologic grade: ovarian carcinoid tumors (Grade 1) and O-NEC (NECs, Grade 3). Unlike other gynecologic sites such as the cervix or uterus—where the term “carcinoid” has been replaced by NET—the ovary retains “carcinoid” due to its unique origin, often linked to monodermal teratomas like dermoid cysts. Ovarian carcinoids are usually well-differentiated and slow-growing tumors [4]. 

In contrast, O-NECs which include small cell and large cell subtypes, represent a far more aggressive subset of ovarian tumors. Small cell carcinoma of the ovary (SCCO) exists in two distinct histologic entities: the pulmonary type (SCCOPT) and the hypercalcemic type (SCCOHT) [4, 7]. Based on the 2014 WHO classification, SCCOPT is classified under miscellaneous ovarian tumors and is recognized as part of the neuroendocrine neoplasm spectrum. In contrast, SCCOHT is no longer categorized within the NEC; instead, it is considered a genetically distinct entity with closer molecular alignment to rhabdoid-like tumors [4]. Therefore, the terminology “small cell neuroendocrine carcinoma of the ovary” (SCNEO) in this study specifically refers to SCCOPT. Furthermore, large cell neuroendocrine carcinomas of the ovary (LCNEO) are now considered synonymous with undifferentiated non-small cell neuroendocrine carcinoma of the ovary (NSCNEO), and like their small cell counterparts, are associated with an aggressive clinical course and poor prognosis [4]. 

The rarity and heterogeneity of O-NECs underscore the urgent need for improved diagnostic clarity and standardized therapeutic strategies in the management of these malignancies. Our review highlights a significant gap in clinical data and evidence-based protocols particularly for O-NEC, which may contribute to suboptimal outcomes. This research presents findings from a thorough literature review, including cohort studies, case series, and case reports, which examine the management and prognosis of O-NEC, mainly emphasizes established therapeutic approaches and related outcomes observed in women diagnosed with this malignancy. Using the available data, we aim to contribute to the understanding and possible improvement of treatment strategies for this rare and challenging subtype of ovarian cancer.

## Materials and methods

### Literature search

We performed a systematic review in accordance with the PRISMA guidelines. This review was registered in the International Prospective Register of Systematic Reviews (PROSPERO, number CRD42024574176). A systematic literature search from the databases in PubMed, Science Direct, Wiley, Springer Link, Google Scholar and Cochrane Central Register of Controlled Trials was performed on August 1st, 2024. We identified 167 citations, and 27 citations were included after screening all abstracts. From the 27 articles, 6 articles were excluded because 5 articles required paid access and 1 article was a preprint, resulting in the inclusion of 21 studies. Review studies, double publications, abstract-only publications, and studies with metastatic neuroendocrine carcinoma to the ovary were excluded. Articles using languages other than English were also excluded. The keywords applied were “Neuroendocrine Carcinoma” OR “Neuroendocrine Tumours” AND “Ovary” OR “Ovarian” AND “Management” OR “Treatment” OR “Therapy” AND “Outcome” OR “Outcomes”.

### Study selection

Articles obtained through the search process underwent relevance screening of titles, abstracts, and full texts by three authors (KIM, ASH, GNAW) independently. Mendeley was utilized as a tool to assist the authors in reading and screening each article, grouping and organizing articles, detecting duplicate publications, and managing references and citations. Any discrepancies were resolved through consensus after assessment by a fourth author (ABH).

Studies included in this review were those that described therapies—including surgical interventions, chemotherapy, and radiation—and patient outcomes following treatment for O-NEC. The inclusion criteria encompassed original articles and case reports published in either English or Indonesian to mitigate potential bias arising from translation processes. Conversely, this review excluded studies in the form of literature reviews, commentaries, letters to the editor, non-full-text articles, and unpublished or non-peer-reviewed materials.

### Data extraction

Three authors (FE, DS, AK) independently used a predetermined data extraction form to collect and record data from the selected studies. For each study, the extracted data included study characteristics (first author and year of publication), study design (type of study, data source, number of cases, incidence), and case characteristics (FIGO stage, histology, IHC examination results). Additionally, we collected data on therapeutic modalities (type of surgery, chemotherapy, radiation, and agents used), outcomes (survival rate and survival duration), and follow-up results of patients who had undergone therapy.

### Quality assessment

**Fig. 1:**
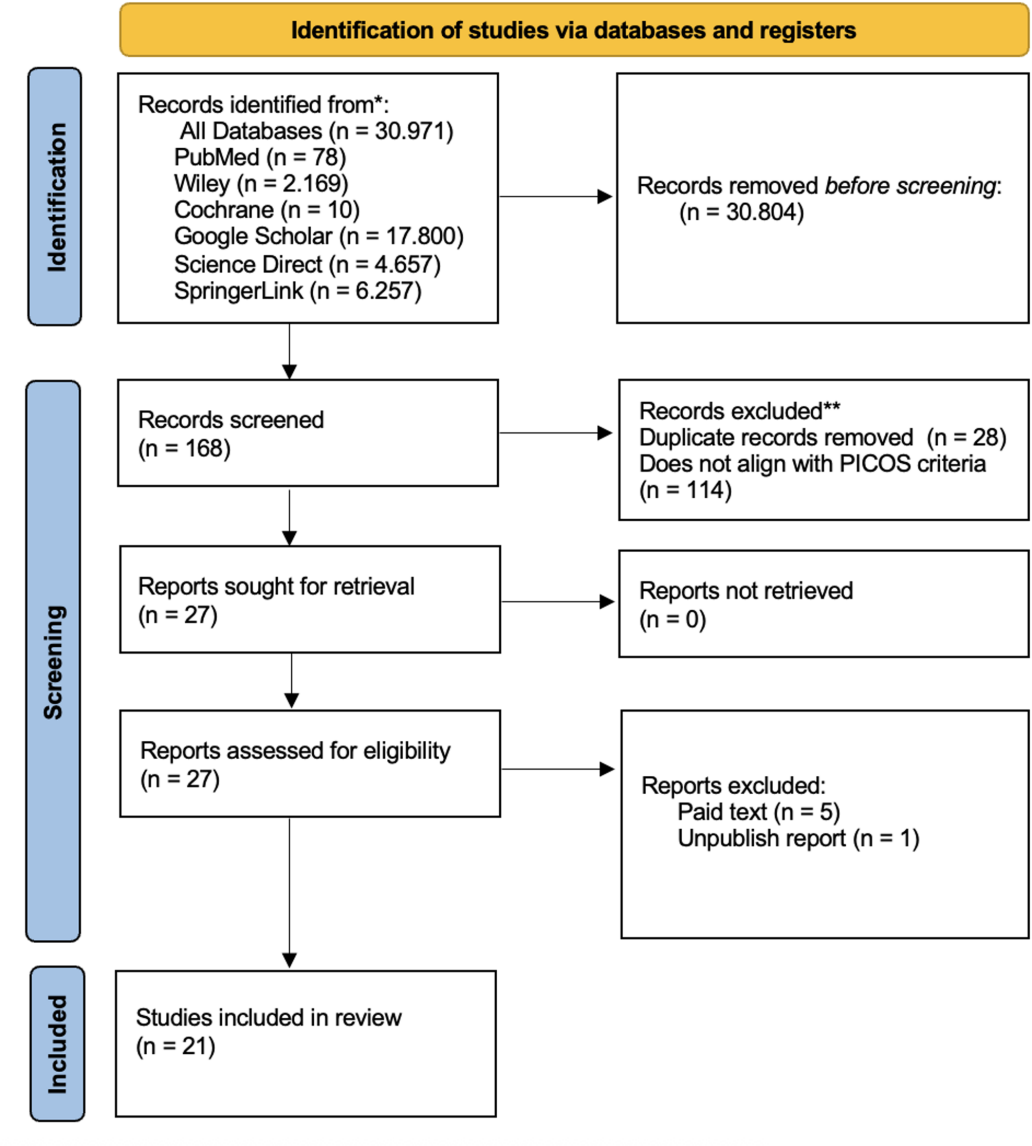
PRISMA diagram

The quality of the included articles was assessed by three authors (SS, YMH, NCK) using the Joanna Briggs Institute (JBI) critical appraisal critical review checklist which is suitable for assessing the quality cohort and case–report studies. The checklist can be accessed at https://jbi.global. The stages of searching and selecting articles are summarized in Fig. 1. PRISMA diagram.

## Results

The studies included in this systematic review were gathered from a comprehensive literature search conducted on August 1st, 2024, across databases including PubMed, Cochrane Central Register of Controlled Trials, Springer Link, Wiley, and Science Direct. This search identified 168 citations, of which 21 were selected for analysis after thorough screening of all abstracts [8–28]. Excluded from the review were studies that were review articles, duplicate publications, abstracts only, and a study focused on women with neuroendocrine tumors metastatic to the ovary. Figure 1 shows a flow diagram of the literature search.

Of these 21 studies reviewed, we found 6 retrospective cohort studies, 12 case reports and 3 cases series. No prospective study or interventional trial was identified. Only 3 studies reported on ≥ 100 patients with O-NEC describing 431 [10], 219 [9], and 217 [12] cases, respectively. In summary, 923 cases of O-NEC have been reported in the literature. The respective incidences given in these studies were 219 of 23.917 cases (0.9%) [9], 7 of 27 cases (26%) [12] and 3 of 12 cases (25%) [13] or a pooled data.

The most common histological subtype of O-NEC was SCNEO, however, it differs by only one case compared to LCNEO. In the identified cases of O-NEC and ovarian carcinoid tumors, the subtypes SCNEO, LCNEO, carcinoid, and unknown accounted for 369 of 923 cases (40%), 368 of 923 cases (39.8%), 122 of 923 cases (13.2%), and 64 of 923 cases (7%), respectively.

According to the FIGO classification, ovarian cancer is categorized into four stages. In this study, cases were distributed as follows: Stage I-II comprised 260 of 923 cases (28%), Stage III-IV made up 479 of 923 cases (52%), and 184 cases (20%) had an unknown stage (Table 1). The percentage cases of stage III-IV was notably higher than that of stage I-II. This stage distribution at initial diagnosis provides valuable insights into how the disease typically presents within the studied population.

**Table 1:** Clinical, Histopathological, and Immunohistochemical Features of O-NEC

Author	Publication date	Study Design	Number of cases	Incidence	Stage	Small cell/large cell/carcinoid/ unknown	IHC (+/-)
Pang L, et al.	2021	Retrospective	431	-	I = 159 II = 23 III = 101 IV = 148	124/130/118/59	-
Pang L, et al.	2022	Retrospective Cohort	219	219/23.917	I-II = 10 III-IV = 113 unknown = 96	0/219/0/0	-
Pang L, et al.	2022	Retrospective	217	-	I: 31 II: 9 III: 41 IV: 53 Unknown: 83	217/-/-/-	-
Sehouli, et al.	2016	Retrospective	11	-	FIGO 1: 5 2: 1 3: 3 4: 2	2/2/2/5	**NEC** CgA (*n* = 8) (5/3) Syn (*n* = 7) (5/2) CD56 (*n* = 6) (5/1) NSE (*n* = 3) (2/1) **Others** CK7 (*n* = 8) (7/1) CK20 (*n* = 3) (2/1)
Pang L, et al.	2021	Retrospective	7	7/27	I: 2 II: 1 III: 3 IV: 1	7/0/0/0	**NEC** CD56 = 6 SYN = 5 NSE = 3 CgA = 3 **Ca Ovary** P53 = 1 CK = 3 TTF-I = 4 P40 = 4
Xing XY, et al.	2024	Retrospective	3	3/12	IA, IIIB, IIIC	-/3/-/-	**NEC (3)** CD56 (+): 3 Cga (+): 3 Syn (+): 3
Yadav R, et al.	2022	Case report	1	-	III	0/1/0/0 (only large cell)	**NEC** CD56 (+) NSE (+) **Ca Ovary** PR (+) P53 (+)
Gupta P. et al.	2021	Case report	1	-	IA	0/1/0/0	**NEC** CgA: (+) strong NSE: (+) strong **Ca Ovary** Epithelial membrane antigen (-) WT1 (-) inhibin: (-)
Ashrafganjoei T, et al.	2016	Case report	1	-	I		-
Peng X, et al.	2020	Case report	1	-	III	0/1/0/0	**NEC** CD56 (+) NSE (+) P16 (+) CgA: (-) **Ca Ovary** P53 (+) WT-1 (-) CK7 (-) CK20 (-) ER (-) PR (-) (CK)-P: (+)
Tartaglia E, et al.	2008	Case report	1	-	IIIC	0/1/0/0	**NEC** NSE (+) CD56: (+)
Li Y, et al.	2022	Case report	1	-	-	1/0/0/0	**NEC** CD56 (+), CgA (+) Syn (+) **Ca Ovarium** PAX-8 (-) Vimentin (-) WT-1 (-).
Xing Y, et al.	2024	Case report	1	-	-	0/1/0/0	**NEC** CgA(-) Syn(-) CD56(- her2(1+)
Lin C.H, et al.	2014	Case report	1	-	IV	-/1/-/-	**NEC** CD56 (+) Chromogranin A (+) NSE (+) **Ca Ovary** CK7 (-) CK20 (-) vimentin (+)
Yang X, et al.	2019	Case report	1	-	III	-/1/-/-	**NEC** Syn (+) CK (+) **Ca Ovary** WT-1 (+) Vimentin (+) CgA (+)
Peng X, et al.	2020	Case report	1	-	III	-/1/-/-	**NEC** CD56 (+) neuron-specific enolase (NSE) (+) CgA (-) **Ca Ovary** P53 (+) P16 (+) (CK)-P (+) WT-1 (-) CK7 (-) CK20 (-) ER (-) PR (-)
Herold N, et al.	2018	Case report	1	-	IV	-/1/-/-	**NEC** Syn (+) CD56 (+) CgA (+) **Ca Overy** CK20 (-) TTF (-)
Tsuyoshi H, et al.	2019	Case report	1	-	IIIC	-/1/-/-	**NEC** Syn (+) CD56 (+)
Feng BJ, et al.	2023	Case series		-	II: 1	1/-/2/-	**NEC** Syn: 2/3 CgA: 2/3 CD56: 3/3 **Others** inhibin: 0/3 CKPan: 3/3
Ki E, et al.	2014	Case series	3	-	I, IA, IIB	-/3/-/-	**NEC** Syn: 0/3 CgA: 2/3 NSE: 3 /3
Harrison ML, et al.	2006	Case Series	17	-	I: 12 III: 4 Unknown: 1	17/-/-/-	**-**

The immunohistochemical markers utilized as diagnostic criteria for O-NEC include synaptophysin, chromogranin A, CD56, and NSE. In our analysis, the immunoreactivity results were as follows: synaptophysin demonstrated positive staining in 84% of cases (21/25), while chromogranin A was positive in 64.2% of cases (18/28), CD56 showed positivity in 69% of cases (20/29), and NSE was expressed in 77.7% of cases (14/18). Table 1 demonstrates the positive reactivity of immunohistochemical staining of O-NEC in the study.

Table 2 presents the treatment modalities and outcomes. The most frequently used primary treatment for O-NEC was surgical intervention alone. Other treatment options included surgery combined with chemotherapy, surgery followed by chemoradiation, surgery followed by radiation, chemoradiotherapy, and chemotherapy alone. An analysis of the 21 studies included in the review showed the following distribution of treatment modalities. In total, 755 therapeutic interventions were recorded surgical intervention alone accounted for 171 out of 487 cases (35%), a combined surgical and chemotherapeutic approach for 156 out of 487 cases (32%), surgery followed by chemoradiotherapy for 23 out of 487 cases (5%), concurrent chemoradiotherapy for 8 out of 487 cases (2%), chemotherapy as the sole modality for 63 out of 487 cases (13%), radiotherapy alone for 4 out of 487 cases (1%), no treatment 62/487 (12%), and unknown treatment in 436 cases. The published studies did not include any retrospective or prospective comparisons of the effectiveness of surgery-based, chemotherapy-based, and radiotherapy-based treatment approaches within similar disease stages.

**Table 2:** Treatment Modalities, Surgical Approaches, Chemotherapy Regimens, and Outcomes in Patients with O-NEC

Author	Publication date	Number of cases	Treatment Modalities (initial)	Treatment Modalities (recurrence)	Surgery	Surgery (recurrence)	Chemotherapy regimens	Chemotherapy regimens (recurrence)	Follow Up
Pang L, et al.	2021	431	S: 162 S + CTX: 116 S + CTX + RT: 17 CTX: 62 RT + CTX: 8 RT: 4 No treatment: 62	-	-	-	-	-	4.5 years, 22 out of 34 patients showed no signs of recurrence, and the disease remained static; 12 out of 34 patients had metastases, 5 patients received somatostatin analogs or chemotherapy, and 8 patients (23.5%) died of the disease
Pang L, et al.	2022	219	S: 141 CTX: 120 RT: 7	-	-	-	Etoposide and cisplatin/ carboplatin or paclitaxel and carboplatin	-	-
Pang L, et al.	2022	217	unknown	-	unknown	-	-	-	-
SEHOULI, et al.	2016	11	S + CTX: 6 S: 5	-	Bilateral salpingo-oophorectomy: 10 unilateral SO: 1 Hysterectomy: 9 Omentectomy: 9 pelvic/paraaortic lymphonodectomy: 5 partial colon resection: 5 appendectomy: 5	-	Carboplatin/Paclitaxel: 4 Cisplatin: 1 Cisplatin/Etoposide: 1 none: 5	-	-
Pang L, et al.	2021	7	S + CTX: 7	-	UAd (Unilateral Adnexedtomy): 1 SS (Staged Surgery): 2 CS (Cytoreductive surgery): 2 induced + CS (Cytoreductive surgery): 1 NBAC (Needle Biopsy of the Abdominal Cavity): 1	-	Etoposide/Cisplatin (6 cycle): 3 Etoposide/Cisplatin (4 cycle): 1 Bleomycin/Etoposide/Cisplatin (6 cycle): 2 Paclitaxel/Carboplatin (1 cycle) + Bleomycin/Etoposide/Cisplatin (1 cycle): 1	-	2 patient still alive 5 patient died (7 m, 30 m, 16 m, 21 m, 3 m)
Xing XY, et al.	2024	3	S + CTX: 3	-	Bilateral salpingo-oophorectomy; Lymph node dissection, total abdominal hysterectomy, omentectomy	-	(1) Etoposide/Cisplatin (6cycle) (2) Paclitaxel/carboplatin (6cycle) (3) Paclitaxel/Carboplatin (6cycle) + PARPi	-	(1) No evidence disease (2) No evidence disease (3) Alive with disease
Yadav R, et al.	2022	1	S + CTX: 1	-	abdominal hysterectomy with bilateral salpingo-oophorectomy with abdominopelvic mass and sigmoid resection followed by reanastomosis and diversion ileostomy.	-	Etoposide	-	After 5 months of primary surgery, contrast-enhanced computed tomography of the chest, abdomen, and pelvis revealed recurrence. She succumbed to her illness 6 months after primary surgery
Gupta P. et al.	2021	1	S + CTX	-	abdominal hysterectomy with bilateral salpingo-oophorectomy	-	Paclitaxel/Carboplatin, (4 cycle)	-	patient is disease-free currently, 28 months post-treatment.
Ashrafganjoei T, et al.	2016	1	S + CTX	-	hystrectomy and right oophorectomy	-	Etoposide/cisplatin (6 cycle)	-	-
Peng X, et al.	2020	1	S + CTX	-	Cytoreductive surgery	-	paclitaxel/carboplatin (5 cycle)	-	Follow-up for 12 months showed no clinical or radiological evidence of disease recurrence.
Tartaglia E, et al.	2008	1	S + CTX	-	Cytoreductive surgery	-	etoposide/cisplatin	-	patient died 2 months after surgery
Li Y, et al.	2022	1	S + CTX	-	laparoscopic total uterine double attachment resection, bilateral ovarian arteriovenous high ligation, abdominal catheterization	-	etoposide/cisplatin (1 cycle) paclitaxel/cisplatin (4 cycle) Illinotecan/apatinib (1 cycle) Carrelizumab/apatinib (2 cycle)	-	The patient was still alive at a follow-up performed two years after surgery. There were no signs of recurrence during the follow-up period.
Xing Y, et al.	2024	1	S + CTX	S + chemo	radical ovarian surgery	pelvic mass resection	Paclitaxel/Carboplatin (6 cycle)	TC 3 cycle, paclitaxel + bevacizumab + pembrolizumab 2 cycle, oral etoposide + anlotinib	Disease progressed after 4 months and After this progression, she was screened for enrollment in a clinical trial in September 2022.
Lin C.H, et al.	2014	1	S	-	total abdominal hysterectomy, bilateral salpingo-oophorectomy, partial omentectomy, and appendectomy. Lymph node dissection was omitted to avoid excessive bleeding	-	-	-	patient died 3 months postsurgery
Yang X, et al.	2019	1	S + CTX	-	total abdominal hysterectomy with left salpingo-oophorectomy, omentectomy, along with removal of pelvic metastases	-	Etoposide/cisplatin (1 cycle) Etoposide (4 cycle)	-	Follow up after 3 months still alive
Peng X, et al.	2020	1	S + CTX	-	Cytoreductive surgery	-	paclitaxel/cisplatin (5 cycle)	-	Follow-up for 12 months showed no clinical or radiological evidence of disease recurrence.
Herold N, et al.	2018	1	S + CTX	-	Abdominal bilateral salpingo-oophorectomy, omentectomy, and deperitonealisation of the pelvis pelvic and paraaortal lymphonodectomy.	-	etoposide/carboplatin (2 cycle) paclitaxel/carboplatin (1 cycle)	-	Until December 2016, there were no signs of recurrent disease.
Tsuyoshi H, et al.	2019	1	S + CTX	-	exploratory laparoscopy	-	Etoposide/Cisplatin	-	patient died 2 months after surgery.
Feng BJ, et al.	2023	3	S: 3	-	(1) total hysterectomy, removal of the right adnexa and left ovarian tumor, left fallopian tube removal, greater omentum removal, appendectomy, and tumor reduction (2) Laparoscopic resection of the left adnexa (3) laparoscopic bilateral adnexectomy	-	-	-	(1) passed away 21 months after the surgery (2) survived for 9 years (3) survived for 10 years,
Ki E, et al.	2014	3	S + CTX: 3	(2) S + chemo	(1) exploratory laparotomy (2) abdominal hysterectomy with bilateral salpingo-oophorectomy, total omentectomy, and pelvic lymph node sampling at the private clinic (3) total abdominal hysterectomy, bilateral salpingo-oophorectomy, pelvic lymph node dissection, para-aortic lymph node dissection, and total omentectomy along with multiple biopsy	(2) debulking operation	(1) etoposide (1 cycle) + Carboplatin (1 cycle) (2) Paclitaxel/Cisplatin (6 cycle) (3) Paclitaxel/Carboplatin	(2) 7 sessions of taxotere 75 mg/m2 every 3 weeks	(1) She died of septic shock after 45 postoperative days. (2) died of multiple organ failure at 17 months after initial diagnosis. (3) She is still healthy 5 months after operation.
Harrison ML, et al.	2006	17	S + CTX: 10 S + CTX + RT: 6 CTX: 1	S: 1	Oophorectomy: 5 Hysterectomy: 7 Bilateral Salpingo-oophorectomy: 6 Salpingo-oophorectomy: 3 Omentectomy: 6 Node Sampling: 2	Omentectomy: 1 Node sampling: 1	Bleomycin/Etoposide/Cisplatin: 3 Cisplatin/Etoposide: 6 Cyclophosphamide/Etoposide/Cisplatin: 1 Carboplatin/Paclitaxel: 6 Cisplatin/Paclitaxel/Etoposide/Carboplatin: 2 Carboplatin: 1	-	Alive without disease ≥ 10 months: 7 Alive without disease 5 moths: 1 Died: 7 Alive with disease 8 months: 1 Alive with disease 16 months: 1

Two studies did not provide individual patient treatment details. The first study did not present detailed data [10], whereas study by Pang L, et al. (2022) reported aggregated treatment data which is out of 219 cases, 141 underwent surgical intervention, 120 received chemotherapy, and 7 cases were treated with radiation therapy [9]. 

Through analysis of 21 studies on O-NEC revealed no standardized chemotherapy regimen. The most used treatments regiment were etoposide/cisplatin (45.4% of studies) [9–28]. Other regiments may include many variations of platinum-based therapies and combinations like bleomycin/etoposide/cisplatin [11, 28]. 

Some studies reported the use of new targeted therapies and immunotherapeutic agents. The combination therapies included irinotecan with apatinib (a VEGFR-2 inhibitor) and carrelizumab (an anti-PD-1 antibody) with apatinib. These indicate an increasing interest in novel approaches for O-NEC treatment [19]. 

From our findings, there is a significant gap in the literature that provide information on treatment protocols for recurrent O-NEC, with only 3 out of 21 studies (14%). In the first case, the patient’s pelvic mass was resected, followed by chemotherapy with Paclitaxel/Carboplatin for 3 cycles, paclitaxel combined with bevacizumab and pembrolizumab for 2 cycles and oral administration of etoposide plus anlotinib. In second case, the patient underwent debulking surgery followed by adjuvant chemotherapy consisting of 7 sessions of docetaxel 75 mg/m² every 3 weeks. And in the last case the patient underwent only omentectomy and node sampling surgery.

Due to the rarity of O-NEC and the limited available data, few studies have comprehensively analyzed survival rates associated with different treatment modalities. Table 3 identified six studies that provided insights into survival outcomes following various therapeutic interventions, published between 2016 and 2024 with a total of 888 cases of O-NEC, with sample sizes varying considerably, ranging from 3 to 431 patients [8–13]. 

**Table 3:** Survival Outcomes in Patients with O-NEC

Author	Publication date	Number of cases	Cancer specific survival duration	progression-free survival duration	OS duration	3 year cancer specific survival rate	3 year free survival rate	3 year OS rate	5 year cancer specific survival rate	5 year free survival rate	5 year OS rate
SEHOULI, et al.	2016	11		12 months	20 months			-		-	-
Pang L, et al.	2021	431							Stage I: 85.6% Stage II: 41.7% Stage III: 21.2% Stage IV: 9.8%	-	Low grade: 93.96% High grade: 7.01%, Stage I: 83.3% Stage II: 30% Stage III: 20.3% Stage IV 9.8%
Pang L, et al.	2022	219	19 months		20 months	38.6%		37.1%	32.3%		28.7%
Pang L, et al.	2021	34		14.71 months	19.28 months		47.6%,	27%		-	-
Xing XY, et al.	2024	3		13 months	19.5 months		-	33.3%		-	27.6%
Pang L, et al.	2022	217			11 months			23.5%			22%

In 2016 Sehouli et al. reported on 11 O-NEC cases. The research demonstrated a progression-free survival (PFS) of 12 months and an overall survival (OS) duration of 20 months [11]. In 2021, Pang et al. conducted a more extensive retrospective study involving 431 O-NEC patients, the study showed significant variations in 5-year OS rates based on tumor grade and stage. Low-grade tumors showed a 93.69% survival rate, while high-grade tumors had a markedly lower rate of 7.01%. Stage-specific 5-year OS rates were 83.3%, 30%, 20.3%, and 9.8% for stages I through IV, respectively. The same study reported 5-year cancer-specific survival (CSS) rates of 85.6%, 41.7%, 21.2%, and 9.8% for stages I through IV [8]. 

Research conducted by Pang et al. (2022) focused on specific O-NEC subtypes. The research is a retrospective cohort of 219 patients with LCNEO with an overall duration of 20 months and a CSS duration of 19 months. The data shows that the 2- or 3-year OS rate was 37.1%. Meanwhile, the 5-year rate dropped to 28.7%, and it also shows that CSS rates for 2 or 3 years were 38.6% and for 5 years were 32.3% [9]. Pang et al. also conducted a similar study in the same year on 217 cases of SCNEO, the OS duration was 11 months, with a 3-year OS rate of 23.5% and a 5-year OS rate of 22% [10]. 

Small Cell Neuroendocrine Carcinoma of the Ovary (SCNEO) has been observed by Pang et al. (2022), and 7 cases identified an OS duration of 19.28 months. The two or three years PFS rate was 47.6%, with a two or three years OS rate of 27% [12]. A recent study conducted by Xing et al. (2024) reported a two or three-year CSS rate of 33.3% and a five-year OS rate of 27.6%. These reports show the aggressive nature of O-NEC and the urgent need for more effective treatment strategies to improve long-term survival outcomes [13]. 

The median OS ranged from 11 to 23.5 months across the studies. Pang et al. reported a median OS of 20 months in their 2021 study of 431 patients [8], while another study by the same author in 2022 found a median OS of 20 months in a cohort of 219 patients [9]. The shortest median OS of 11 months was observed in a study of 217 patients with small-cell neuroendocrine carcinoma of the gynecologic tract [10].

Progression-free survival (PFS) was reported in three studies, with median values ranging from 12 to 14.71 months. The longest median PFS of 14.71 months was found in a study focusing on small-cell carcinoma of the gynecologic tract [12]. The CSS was reported in only one study, with median durations of 19 months [9].

The 3-year OS rate ranged from 23.5 to 37.1% [9, 10]. The 3-year PFS rate was 47.6% in one study [12], while the 3-year CSS rate was 38.6% in another [11]. Five-year survival rates showed considerable variation. The 5-year OS rate ranged from 22 to 28.7% [9, 10]. Notably, Pang et al. found that 5-year CSS rates varied significantly by stage, with 83.3% for stage I, 30% for stage II, 20.3% for stage III, and 9.8% for stage IV [8].

Several studies identified factors associated with survival outcomes. The stage at diagnosis was consistently reported as a significant prognostic factor. Pang et al. found that patients with low-grade tumors had significantly better 5-year CSS rates (93.96%) compared to those with high-grade tumors (7.01%) [8].

The reviewed studies indicate that O-NEC generally has a poor prognosis, with median OS ranging from 11 to 23.5 months. Early-stage disease and low-grade tumors were associated with better survival outcomes. Nevertheless, caution is warranted when interpreting these results due to the retrospective nature of the studies and the heterogeneity in sample sizes and the different NEC subtypes discussed.

## Discussions

In this study, O-NEC relatively has a small incidence [11–17]. Due to the rarity of the disease, the research involving O-NEC also became rare, making it challenging to develop standardized treatment protocols including the diagnostic challenges, treatment strategies, and overall prognosis [13]. Moreover, there are still no effective screening tools available for the general population, and this limitation also has significant economic implications. Over the past decade, various cost-effective strategies for early detection and prevention of ovarian cancer have been explored, given that the cost of treatment per patient with ovarian cancer remains among the highest across all cancer types. For instance, the average initial costs in the first year of treatment can reach approximately USD 80,000, with the final year costs potentially increasing to around USD 100,000 [29]. Current therapeutic strategies for O-NEC have largely adhered to established treatment paradigms for general ovarian malignancies. Therefore, this systematic review is needed to synthesize the findings from various studies to provide a comprehensive overview of the current management and diagnosis protocols for O-NEC [13]. 

This review included significant heterogeneity studies in design, sample size, and methodologies. Most studies are case reports, with only a few retrospective studies and case series available. This diverse types of studies analysis indicate significant variation in involved type studies, but the high number of individual case reports also implies a relatively low incidence and the rarity of O-NEC, which complicates the ability to derive standardized treatment protocols.

Findings from the 21 included studies were extracted in relation to two key areas: treatment protocols and survival rate of the cases. In this review Sehouli et al. (2016) conducted a retrospective study with 11 cases of O-NEC, while Pang et al. (2021) presenting 431 ovarian NENs cases, Pang et al. (2022) presenting 219 LCNEO cases and 217 SCNEO cases respectively, but still faced challenges due to the diversity in clinical presentation and treatment approaches [8–10]. This variability significantly influences the determination of the required standard therapy protocol, therefore collaborative effort and multicenter studies to consolidate data and improve understanding of this rare malignancy is needed [13]. 

Advanced-stage disease patients are more frequently found in cases of O-NEC compared to those with early-stage disease. Studies by Sehouli et al. (2016), Pang et al. (2021), and Pang et al. (2022) highlight the majority of patients are diagnosed at FIGO stages III and IV, which significantly impacts their prognosis [8–13]. Diagnosis in advanced stages of disease shows the need for clinical awareness improvement strategies. For confirming the diagnosis of O-NEC, a comprehensive diagnosis is needed, including patient history, physical examination, laboratory test, and imaging studies. One of the tests that serves as the gold standard is Immunohistochemical (IHC) markers. Chromogranin A (CgA), Synaptophysin (Syn), and CD56 are essential for confirming the diagnosis of NEC, as they help differentiate these tumors from other ovarian malignancies. For example, Yadav et al. (2022) utilized these markers to diagnose a case of primary pure LCNEO, emphasizing their diagnostic utility [14].

At present, there are no standardized treatment protocols for O-NEC. In clinical practice, most patients are managed according to treatment guidelines for epithelial ovarian cancer. This typically involves extensive surgical resection followed by adjuvant chemotherapy, which remains the cornerstone of therapy for newly diagnosed high-grade serous epithelial ovarian cancer. In situations where primary debulking surgery is not feasible either due to poor patient performance status or the complexity of achieving optimal cytoreduction, neoadjuvant chemotherapy (NACT) may serve as an appropriate alternative approach [30]. 

Surgical treatment remains the option of treatment for O-NEC. Common surgical procedures include bilateral salpingo-oophorectomy (BSO), hysterectomy (HE), and omentectomy. Based on research conducted by Sehouli et al. (2016), various surgical approaches depending on the disease extent, and if the patient wanted fertility preservation, unilateral salpingo-oophorectomy was a consideration [11]. After surgical resection, adjuvant chemotherapy is typically administered to manage residual disease and reduce the risk of cancer recurrence.

As previously mentioned regarding the use of chemotherapy in O-NEC, which can be as sole modality or as a combination therapy, we identified several chemotherapy protocols that have been employed in clinical practice, albeit with varying frequencies. Chemotherapy platinum-based regimens, particularly those including carboplatin and cisplatin, combined with taxanes chemotherapy regimens are commonly one of the options. Sehouli et al. (2016) reported the use of carboplatin and paclitaxel in four cases, while Pang et al. (2022) indicated the effectiveness of etoposide in combination with platinum agents [9]. These regimens are frequently chosen for chemotherapy in gynecologic cancers, particularly ovarian cancer, and to date there are still limitations in the use of chemotherapy regimens for NEC-type cancers [8]. Etoposide/Cisplatin is the most common combination of chemotherapy regimens reported in 10 out of 21 studies (45%) followed by the paclitaxel/carboplatin combination, which was documented in 8 out of 21 studies (36%). The variety of regimens also may reflect the rarity of the disease and the lack of systematic population-based research or registration data [8]. 

Immunotherapy and targeted therapy, which have been used in several studies in this review, indicate that efforts have begun to develop treatments for patients with O-NEC. Some studies reported the use of targeted therapies and immunotherapy agents including irinotecan as known as topoisomerase inhibitor, combination with apatinib, a tyrosine kinase inhibitor targeting VEGFR-2). Furthermore, the use combination of cerrelizumab and apatinib was also noted. Cerrelizumab is an anti-PD-1 monoclonal antibody.

Recent strategies have also explored the combination of PARP inhibitors with immune checkpoint inhibitors (such as anti-CTLA-4 and PD-1/PD-L1), particularly in tumors with homologous recombination (HR) deficiency, which tend to exhibit higher neoantigen loads and enhanced immune response. Revythis A, et al. stated “the rationale for combining PARP inhibitors with immunotherapy is partly based on the immunogenic effects of HR deficiency and the immune modulatory roles of PARP inhibition itself.” [31].

This heterogeneity in treatment approaches highlights the ongoing challenge in establishing a gold standard for O-NEC management and the need for further research to determine the most effective chemotherapy or targeted therapy regimens to specific O-NEC subtypes or molecular profiles.

Due to lacking of gold standard management of O-NEC, managing recurrent ovarian NEC poses significant challenges. With many studies not providing detailed regimens, the data on specific chemotherapy regimens for recurrent disease are sparse. Sehouli et al. (2016) did not specify the chemotherapy agents for patient with recurrent disease, thus highlighting the need for more detailed reporting and research, especially in this area [11]. 

Patients with O-NEC generally have a poor prognosis with high rates of recurrence and mortality. Among the 21 studies reviewed, three studies did not provide information on patient follow-up outcomes post-treatment. Of the remaining 18 studies, eight studies reported patient mortality, with a total of 26 deaths following treatment. The patients reported survival times ranging from a few months to several years, depending on the disease stage and treatment purposes. The prognosis variability shows the aggressive nature of O-NEC and the critical need of effective treatment strategies. Although many patients were reported to have died, several studies still demonstrated positive outcomes in patients with O-NEC following treatment. For example, Pang et al. (2021) reported that 22 out of 34 patients had no signs of recurrence after 4.5 years, indicating the potential for long-term survival in select cases [10]. These findings highlight the need for robust prognostic markers to guide treatment decisions and improve patient outcomes. In recent studies, proteomic technologies, such as mass spectrometry and protein array analysis, are crucial in identifying molecular biomarkers and understanding adaptive responses to therapy. These advancements can uncover new therapeutic targets, reduce drug resistance, and improve patient outcomes in ovarian cancer [32]. 

Median survival of O-NEC remains poor, ranging from 11 to 20 months for OS, and 12 to 14.71 months for median PFS [8–13]. This aligns with previous literature describing NEC as an aggressive entity with unfavorable outcomes by Fagganio, et al. (2012) [33]. The variation of OS and PFS in O-NEC across studies may be attributed to differences in patient population, specific NEC subtypes included, and management strategies employed. The relatively short interval between disease progression and death as evidenced by the difference between PFS and OS highlights the limited efficacy of salvage treatments and the need for more effective second-line therapies.

The poor prognosis associated with O-NEC, particularly in advanced stages, shows the importance of a multidisciplinary approach to management. The significant survival advantage observed in early-stage disease supports aggressive surgical efforts when feasible. However, the optimal adjuvant treatment regimen remains unclear and warrants further investigation. The notable influence of tumor grade on survival suggests that this factor should be carefully considered in treatment planning.

Stages at diagnosis have an important role as a critical prognostic factor across multiple studies. The striking difference in 5-year CSS rates between stage I (83.3%) and stage IV (9.8%) disease emphasizes the importance of early detection. However, the rarity of O-NEC and its nonspecific symptoms make early diagnosis challenging. The patients with high tumor grade also appears to significantly influence outcomes, with low-grade tumors associated with markedly better 5-year CSS rates compared to high-grade tumors (93.96% vs. 7.01%) [8]. This substantial difference suggests that tumor grade also should be a key consideration in treatment planning and prognostic discussions with patients.

The survival outcomes observed in this review are generally poorer than those reported for more common ovarian malignancies such as high-grade serous carcinoma [34]. This difference highlights the distinct biological behavior of ovarian NEC and the need for tailored management strategies.

From this review, six retrospective studies all included have a significant limitation, the heterogeneity in sample sizes ranging from 3 to 431 reflects the rarity of O-NEC but also limits the generalizability of some findings, particularly from smaller studies. Future research should focus on prospective, multi-institutional studies to better characterize the natural history of O-NEC and evaluate treatment efficacy. Molecular profiling studies could help identify potential therapeutic targets and improve our understanding of the biological underpinnings of this disease.

This systematic review highlights its poor prognosis and the significant impact of stage and grade on survival outcomes. While the retrospective nature of the included studies limits definitive conclusions, these findings provide valuable insights to guide clinical decision-making and future research efforts. Continued investigation into novel therapeutic approaches and improved diagnostic strategies is crucial to enhance outcomes for patients with this challenging malignancy.

## Conclusion

Ovarian neuroendocrine carcinoma (O-NEC) represents a rare and heterogeneous group of malignancies with diverse histopathological subtypes, variable immunohistochemical profiles, and a generally poor prognosis, especially in high-grade forms. Early-stage disease may benefit from surgical resection alone, but in the advanced-stage disease require multimodal treatment, including surgery and adjuvant chemotherapy like platinum-based chemotherapy. Immunohistochemical markers such as synaptophysin, chromogranin A, and CD56 remain critical for accurate diagnosis and differentiation from other ovarian neoplasms. Given the limited number of cases and the predominance of retrospective studies and case reports, standardized diagnostic and therapeutic strategies are lacking. Multicenter prospective studies and the establishment of centralized registries are urgently needed to better understand disease biology, optimize treatment algorithms, and improve long-term outcomes for patients with O-NEC.

## Electronic supplementary material

Below is the link to the electronic supplementary material.

Supplementary Material 1

## Data Availability

No datasets were generated or analysed during the current study.
